# Comparison of Bond Strength in the Different Class of Resin Cements to Cast and CAD/CAM Co-Cr Alloys

**DOI:** 10.1155/2021/7843979

**Published:** 2021-12-26

**Authors:** Leyla Sadighpour, Azam S. Mostafavi, Maryam Pirmoradian, Fatemeh Alipuryalda

**Affiliations:** ^1^Dental Research Center, Dentistry Research Institute, Department of Prosthodontics, School of Dentistry, Tehran University of Medical Sciences, Tehran, Iran; ^2^Prosthodontics CCP Clinic, Department of Clinical Science, Faculty of Dentistry, University of Toronto, Toronto, Canada; ^3^Department of Dental Biomaterials, School of Dentistry/Research Center for Science and Technology in Medicine, Tehran University of Medical Sciences, Tehran, Iran; ^4^Private Practice, Tehran, Iran

## Abstract

**Objectives:**

Despite the widespread use of resin cements in cementing dental restorations, their bond strength to CAD/CAM base metal alloys is not widely studied. This study aimed to evaluate the microshear bond strength (*μ*SBS) between cobalt-chrome (Co-Cr) alloys fabricated using casting or CAD/CAM methods with three types of resin cements.

**Materials and Methods:**

Fifty Co-Cr blocks were prepared with CAD/CAM or casting technique. Specimens were divided using primer or not and bonded to three types of resin cements: Panavia F2, RelyX Unicem, and Duo-Link. The differences between the mean *μ*SBS values were analyzed using the two-way ANOVA test and Tukey analysis (*α* = 0.05). The mode of failure was evaluated using a stereomicroscope. In addition, the specimens were examined by scanning electron microscopy (SEM) based on two received signals: backscattered electrons (SEB) and secondary electrons (SEs). One intact alloy specimen in each group was analyzed by energy-dispersive X-ray spectroscopy (EDX).

**Results:**

Most of the specimens in the no-primer group were prematurely debonded. Statistical analyses showed that the interaction between the alloy substrate and cement type was significant (*p*=0.001). The bond strength of Panavia F2 was significantly higher than Duo-Link in the CAD/CAM group (*p*=0.001). SEM evaluation confirmed the difference in grain structures, while EDX showed no remarkable difference in the chemical composition of the alloy substrates.

**Conclusion:**

Alloy fabrication technique may influence the bond strength of resin cements. In the CAD/CAM group, cement containing MDP molecules exhibited higher strength than the etch-and-rinse one.

## 1. Introduction

Despite the advancement of all-ceramic systems with superior esthetic and favorable strength, dental alloys are extensively used in numerous restorations, including extracoronal metal ceramics and implant superstructures resin-bonded partial fixed dental prostheses. Dental alloys can be classified according to their contents (noble or base metals). For decades nickel-chrome (Ni-Cr) alloys were commonly used for dental-fixed partial denture fabrication. However, there is health concern due to a high number of allergies to Ni reported among the population [1]. Recently, Co-Cr alloys gained attention as a substitute to Ni-Cr alloys. These alloys have a high elastic modulus and less density leading to less weight of the prostheses. Furthermore, Co-Cr alloys are more resistant to corrosion and tarnish rather than Ni-Cr alloys [2]. The main elements in Co-Cr alloys are cobalt, chromium, and molybdenum, plus several trace elements such as niobium, tungsten, and manganese. Small changes in main and trace elements could affect an alloy's working properties [3]. In addition, Co-Cr alloys could be employed to fabricate dental restoration utilizing different CAD/CAM-based techniques, such as direct milling and soft metal milling following by the postsintering process [4]. The prefabricated blanks of materials produced under controlled conditions are believed to have superior properties to their conventional counterparts [3, 4]. Longevity of extra- and intracrown indirect restorations partly depends on the choice of luting cement. Cements seal the gap between the restoration and tooth structure, preventing recurrent caries and supporting periodontium health around the restorations [5]. Resin cements have become superior luting agents in dental clinics due to several characteristics, including adhesion to tooth and a wide range of dental materials, high tensile and compressive strength, and low solubility in the oral environment. Moreover, adhesive cements are preferred in the presence of a short clinical crown or an overtapered preparation [6]. Additionally, in resin bonding fixed partial dentures, adhesive cements provide a profound source of retention. Clinical studies revealed that despite the high survival rate of resin-bonded bridges, technical complications such as debonding are frequent over 5 years [7]. Micromechanical and chemical surface treatments have been advocated to improve the bond strength of resin cements to dental alloy. Thus, primers have been developed with functional monomers such as thiophosphoric acid derivatives (MEPS), 6-methacryloyloxyhexyl 2-thiouracil-5-carboxylate (MTU-6), 6-(4-vinylbenzyl-n-propyl) amino-1,3,5-triazine-2,4-dithiol (VBATDT), and 10-methacryloyloxydecyl dihydrogen phosphate (10-MDP) that can bond chemically to metal oxides on the outer surface of the alloys [8, 9]. Accordingly, resin cements may be classified according to their adhesive scheme; Total-etch resin cements require separate bonding procedures (phosphoric acid etch and application of a dentin bonding system) and pretreatment of intaglio surface of restorations [10]. The major drawback of total-etch resin cements is that multiple application steps and techniques are required, and the sensitive procedures may compromise the bonding quality. Self-etch resin cements employ a self-etch primer, and the mixed cement is used over the primed tooth surface. Self-etch resin cements consist of acidic functional monomers such as 10-MDP capable of etching and bonding to a variety of dental substrates, including teeth, ceramics, and metals [11]. Contrary to the above groups, self-adhesive resin cements require no treatment on the tooth surface and many restorative materials. Self-adhesive cements consist of functional acidic monomers that can chemically bond to inorganic filler and metal oxides on the surface of restorative materials. Thus, self-adhesive cements gain attention due to their simplified application (no separate adhesive and cementation steps) [12].

Basically, metal primers were designed to promote adhesive bonding to noble alloys, and their use with base metals is controversial. Di Francescantonico et al. [13] examined the bond strength of resin cements to Ni-Cr and Co-Cr alloys and found that primers' application did not increase the bond strength. Hattar et al. [14] compared shear bond strength of self-adhesive resin cements to a Co-Cr alloy with no surface treatment but airborne particle abrasion. The bond strength of most groups was less than 10 MPa which is not assumed adequate in the clinical scenario [15]. On the contrary, Nima et al. [8] suggested using combined metal primers and adhesives to improve the bond strength of resin composite to Ni-Cr alloy. Likewise, Choo et al. [16] found that combined use of MDP and VBATDT had a positive impact on the resin bond strength to Co-Cr and Au-Ag-Pd alloys. Abreu et al. [17] showed metal primer application enhanced tensile bond strength to both base and noble metal. Generally, an oxide layer is readily available on the surface of base metal alloys. It is assumed that heat treatment and fabrication method can affect the thickness and structure of this layer, which is vital to the bonding process [3]. Accordingly, CAD/CAM alloys may differ in their bonding characteristics to resin cements. Various surface treatments have been recommended to enhance bond strength of resin to metal alloys, such as airborne-particle abrasion, tin plating, silicoating, and applying metal primers [17]. Therefore, the purpose of the present study was to evaluate the bond strength of Co-Cr alloys created using the casting or CAD/CAM methods with three types of resin cements in the presence or absence of a metal primer. The null hypothesis was that neither the type of alloy nor choice of cement would influence the bond strength between the Co-Cr alloy and resin cement.

## 2. Materials and Methods

A power analysis was performed based on the results of a previous study [15]. The sample size was calculated as at least 20 specimens per group for a significance level of *α* = 0.05 and power of 0.82. However, considering the possibility of specimen loss, it was decided to fabricate 25 for each group. Rectangular-shaped Co-Cr alloys were fabricated (10 × 15 × 2 mm) by two methods: casting and CAD/CAM. For the casting group, rectangular-shaped resin specimens (Ceramill PMMA, Amann Girrbach GmbH) were milled (Ceramill Motion II, Amann Girrbach GmbH) and invested using a phosphate-bonded speed investment (Giroinvest super, Amann Girrbach GmbH). The specimens were then preheated following the manufacturer's instructions and cast in a Co-Cr alloy (Girobond NB, Amann Girrbach GmbH) using an induction casting machine (Heracast IQ, Kulzer GmbH). The investments were bench cooled, divested, and cut from sprues. They were further polished in a polishing machine using 600- and 1200-grit sandpaper. Finally, they were air-abraded using 110-*µ*m alumina powder (1 bar of pressure for 1 minute) and steam cleaned with distilled water for 1 minute.

For the CAD/CAM group, Co-Cr blanks (Ceramill Sintron, AmannGirrbach GmbH) were cut in rectangular-shaped specimens, 11% larger than casting specimens to compensate for sintering shrinkage, and sintered under argon gas in a Ceramill Argotherm furnace (Co-Cr sintering furnace, Amann Girrbach GmbH) according to the manufacturer's instructions. The polishing process was performed as described for the casting group. A *μ*SBS test with wire was performed as described in the study by Di Francescantonio et al. [13] Cast and CAD/CAM specimens were divided into two groups for application of primer and no primer. In primer groups, a metal primer (Alloy Primer, Kuraray Noritake Dental Inc.) was applied to the surface of each rectangular specimen. Subsequently, three cements: a dual cure self-adhesive resin cement (RelyX Unicem, 3M Deutschland GmbH), a dual-polymerized self-etch resin cement (Panavia F2, Kuraray Noritake Dental Inc.), and a dual-polymerized total-etch resin cement (Duo-Link, Bisco Inc.) were applied at three points on the specimens' surfaces as follows. Plastic tubes (0.8 mm internal diameter, 1.5 mm length; Tygon, TYG-030, Small Parts Inc.) were placed over the alloy specimens' surface. The resin cements were mixed according to the manufacturers' recommendations, and each tube was filled with one type of cement, light polymerized at 1100 MW/cm2 (Bluephase, Ivoclar Vivadent Inc.) for 20 seconds on each surface and stored in an incubator at 37°C for 24 hours. No-primer groups were treated similarly. To perform the *μ*SBS test, specimens were mounted on the lower part of a universal testing machine (UTM, Zwick Z050, ZwickRoell Co.). A 0.3 mm stainless steel wire was attached to the moving part of the UTM with the aid of a designed jig, looped around the bonded interface, and pulled at a speed of 0.5 mm/min until failure occurred. The *μ*SBS was obtained by dividing the failure load over the cross section of each resin cement cylinder. Additionally, specimens were observed with a stereomicroscope (×40, SZX 12, Olympus Co.) and SEM (Mira II LMU, Tescan Co.) to evaluate failure mode, classified as adhesive (interface failure), cohesive (failure within the cement layer), or mixed. Alloy bonded surfaces were also examined using SEM based on the backscattered electron (SEB) and secondary electron (SE) signals before bonding and after failure. Additionally, to understand the relationship between bond strength and chemical composition of the substrates resulting from the casting and CAD/CAM methods, specimens were analyzed by energy-dispersive X-ray spectroscopy (EDX). The distribution of the main elements, such as aluminum (Al), Co, Cr, oxygen (O), and silicon (Si), was evaluated by SEM mapping. Three-way ANOVA and the post hoc Tukey's test were used to compare the mean *μ*SBS of the groups using SPSS version 18 (IBM Co., Armonk, NY, USA) with a significance of *α* = 0.05.

## 3. Results

**Figure 1:**
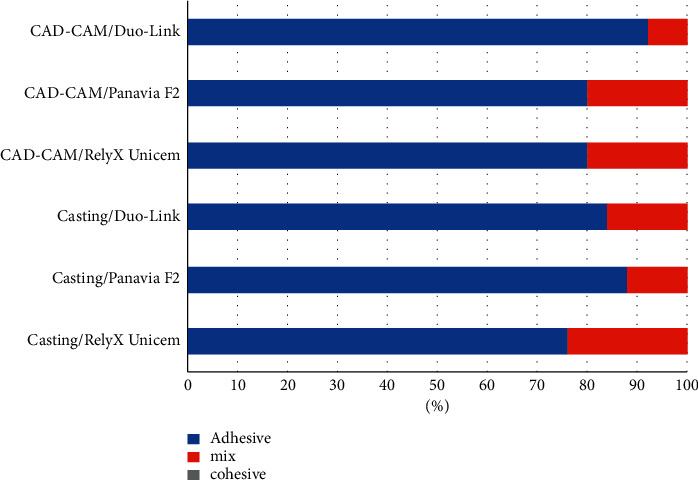
Failure mode (%) of the specimens.

**Figure 2:**
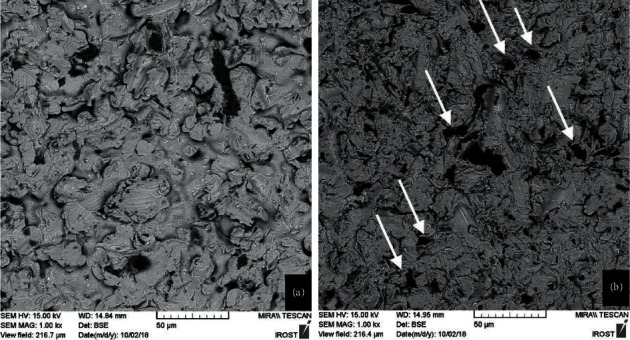
Scanning electron microscopes (SEM) images of metal substrates used in this study. CAD-CAM alloy (a). Casting alloy (b). White arrows indicate areas with defects or impurities in casting specimens.

**Figure 3:**
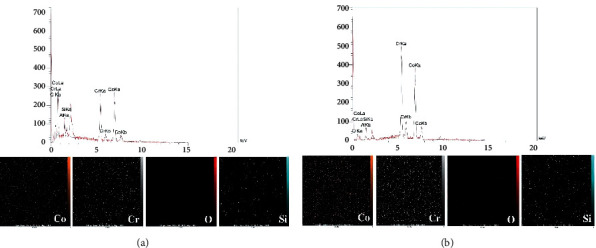
Scanning electron microscopy (SEM) elemental mapping and energy-dispersive X-ray spectroscopy (EDX) spectrum of casting alloy specimens (a) and CAD/CAM alloy specimens (b).

In the no-primer group, all specimens cemented with Duo link were prematurely debonded before test. Specimens in Panaiva F2 and RelyXUnicem groups either prematurely debonded or achieved very low bond strength (<3 MPa). Therefore, no primer groups were excluded from further analysis to avoid the adverse effect on the study power. Mean *μ*SBS values of test groups are presented in Table 1. Two-way ANOVA, showed a significant interaction effect for the two factors (cements and alloys) on the bond strength (*p*=0.001). In the casting group, no significant difference was detected among the cements (*p* > 0.05). However, in the CAD/CAM group, the mean bond strength of Panavia F2 (14.72 ± 4.80 MPa) was significantly greater than that of the Duo-Link cement (10.13 ± 3.71 MPa) (*p*=0.001) and comparable with RelyX Unicem (12.28 ± 3.94 MPa) (*p* > 0.05). Comparing the casting and CAD/CAM groups, Panavia F2 showed a significantly higher bond strength in the CAD/CAM group (*p*=0.001). All failure modes were classified as either adhesive or mixed, with no difference between test groups (Figure 1). SEM observation with SEB signals showed that the alloys of both groups had almost the same microstructure. Generally, CAD-CAM specimens were more homogenous with less structural irregularities, voids, and flaws. In contrast, casting specimens retained more heterogeneous surface topography with variable grain size and inclusion bodies. In addition, grain sizes were larger with more grain boundaries in the casting group (Figure 2). EDX analysis and SEM mapping also revealed similar elements, with the same distribution in both groups (Figure 3).

## 4. Discussion

According to the findings of the present study, it was shown that *μ*SBS was influenced by the type of Co-Cr alloy and resin cement. Therefore, the null hypothesis was rejected. Investigation of the bond strength of resin cements to dental restorations is essential because of its influence on microleakage, biologic complications, and survival [5]. Bonding between an alloy and a resin cement occurs through micromechanical and chemical retention phenomena. Roughening the alloy surface provides the micromechanical retention, while a chemical reaction is believed to occur between the surface metal oxides and acidic functional monomers of the metal primers and/or resin cements [9]. Accordingly, in the present study, all specimens were air-blasted using alumina particles followed by the application of a metal primer on the assigned specimens. The no-primer groups either lost prior to the test or gained very low bond strength. Similar low strength values have been reported by Hattar et al. [14] when evaluating bond strength of self-adhesive cements to a Ni-Cr alloy without administration of any primer.

The mean bond strength of all three cements in the present study was significantly increased upon application of alloy primer. Alloy primer contains two adhesive monomers, MDP and VBATDT. The former is an organic phosphoric ester capable of bonding to oxides available on the base metal alloys. However, VBATDT, a thione-thiol functional monomer, promotes high bond strength to precious metal alloys. Duolink is a Bis-GMA resin-based cement that contains no adhesive monomer of three cements used in this study. Therefore, the MDP of alloy primer improved the bond strength of this cement to the Co-Cr alloys to a great extent. A similar finding has been reported in the earlier study [17, 18]. On the other hand, MDP is a major adhesive molecule in Panavia F2 formula. Initially, no primer was recommended for bond to base metal alloys. However, our results showed the synergic effect of Panaiva with alloy primer in bonding to the Co-Cr alloys used in this survey. Our finding agrees with Shafiei et al. [19] who examined bond strength of resin cements to a Ni-Cr alloy with several surface treatments. On the other hand, Di Francescantonio et al. [13] found that combined Panavia F2 with alloy primer decreased the *μ*SBS to Co-Cr and NiCr alloys. The contradictory result may be attributed to the difference in alloys and experiment methodology. To the knowledge of authors, no further study was found on this subject. However, Dias de Sueza et al. [20] and Yun et al. [21] also found that bond strength of Panavia F2 to zirconia improved when alloy primer was used. High bond strength to zirconia relies on the chemical reaction of 10-MDP with zirconia oxide. It is believed that bond strength to zirconia is strongly promoted by the accumulative concentration of 10-MDP in the primer and the cements [22–24]. This observation may also contribute to our finding with Co-Cr alloy, although it requires further investigation. Unexpectedly, RelyXUnicem also showed a better result with alloy primer compared to the no-primer group. Acidic phosphate methacrylate monomer in RelyXUnicem is capable of chemical bonding to metal oxides without any pre-treatment. However, in the current study, the bond strength without priming was significantly lower than that with alloy primer. Similar findings were reported by Shafiei et al. [19] on Ni-Cr alloy and Dias de Seuza et al [20], Yang et al. [24] and on zirconia ceramic. Contrarily, Abreu et al. [25] studied the effect of alloy primer application combined with RelyXUnicem resin cement on pull-out strength of Co-Cr coping, and no difference was disclosed. There is some concern about VBATDT monomer in alloy primer that may interfere with the polymerization reaction of resin-based materials containing benzoyl amine peroxide in their initiator systems subsequently may disturb the bond between MDP and resin and/or metal [26]. However, in the present study, bond strength with three cements was significantly improved when the primer was applied. Nima et al. [8], Choo et al. [16], and Abreu et al. [17] reported the improved collective effect of MDP and VBTDA in bond strength to base and noble alloys. Since this subject could have some clinical implications, it deserves further study in the future.

In the current study, Panavia demonstrated significantly higher bond strength in CAD/CAM group. This result could be connected to the CAD/CAM milling block manufacturing process that leads to superior chemical and microstructural homogeneity and proper formation of more homogenous and flawless CrO2 film on the surface [27]. EDX and SEM data obtained in this study also demonstrated fewer irregularities, voids, and flaws in CAD/CAM specimens than casting ones. Nevertheless, in line with an earlier study by Al Jabbari et al. [3], they observed similar distribution of the main elements in both experimental groups. One more contributory factor to higher bond strength in CAD/CAM group is that due to proper annealing thermal pre-treatment, residual stresses in the raw material are avoided [3, 27].

**Figure 4:**
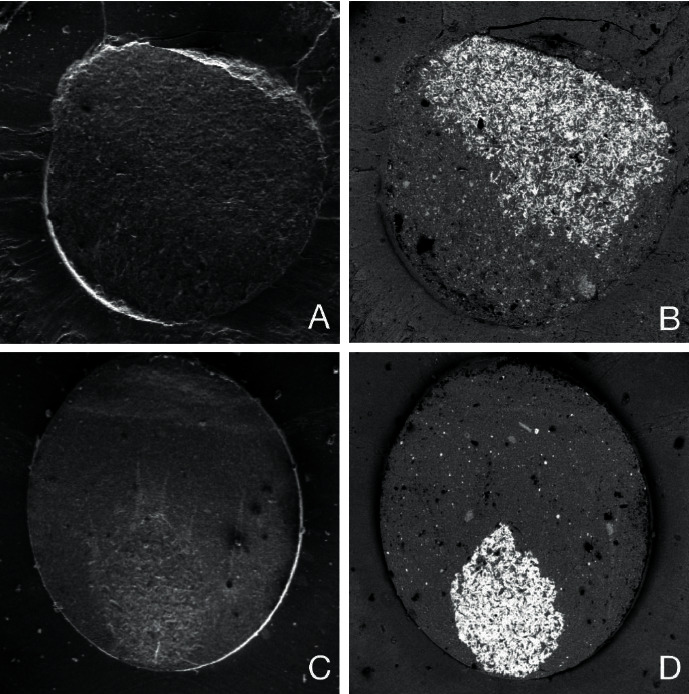
Scanning electron microscopy (SEM) with secondary electron (SE) (a, c) and backscattered electrons (SEB) (b, d) in two different debonded specimens.

In the current study, the *μ*SBS method was used to evaluate the bond strength. It is believed that the debonding force and stress are more evenly distributed across the bonded interface with this method. Moreover, the small size of the specimens rules out failures that may have been caused by random crack propagation within the bonded surfaces [28]. However, specimen҆ dimensions are usually smaller than a real restoration. Hence, this method lacks real clinical resemblance. In the present study, the thermal cycle was not performed to avoid premature specimen loss. Di Francescantonio et al. [13] also ruled out thermal cycling with a similar rationale. Fonseca et al. [29] and Nima et al. [8] observed that the thermal cycle compromised the bond strength of metal to resin. Therefore, the bond strength range achieved in this study overestimated the bond strength in oral circumstances. In the current study, the debonded surfaces were analyzed by SEM with SEB and SE. SEB signals are superior when distinguishing different materials based on their different chemical compositions. For instance, in SEB images, heavier elements like Cr and Co are brighter than carbon and hydrogen (most elements in resin cements are made of methacrylate monomers) (Figure 4). As a mode of failure detection, SEB signals are more reliable, especially when two or three substrates with the same surface topography but different compositions are involved. Most of the failures were in the form of adhesive failure which implies that the bond strength of the Co-Cr alloys was lower than the cohesive strength of the cement. The mean values of bond strength in the cast and CAD/CAM groups were 9.29 ± 5.51 and 14.72 ± 4.80 MPa, respectively. Under clinical conditions, minimum bond strength of 10 to 13 MPa has been considered sufficient to withstand loading in the oral condition [15]. Nonetheless, direct extrapolation to clinical situations may not be possible without further clinical studies due to the complexity of loading and other detrimental factors in the actual condition.

## 5. Conclusion

Within the limitations of this study, it was concluded that the choice of cement and method of alloy fabrication may influence bond strength. In the CAD/CAM group, cement containing 10-MDP molecules (Panavia F2) exhibited higher bond strength compared to etch-and-rinse (Duo-Link) and self-adhesive (RelyX Unicem) cements.

## Figures and Tables

**Table 1:** Mean *μ*SBS and SDs (MPa) in groups with alloy primer.

Groups	PAN			RXU			DUL		
	Min	Max	Mean ± SD	Min	Max	Mean ± SD	Min	Max	Mean ± SD
Casting	2.46	23.59	9.30 ± 5.59^Aa^	6.89	24.15	12.15 ± 5.20^Ba^	2.74	29.20	12.92 ± 7.57^Ba^
CAD/CAM	4.80	23.15	14.72 ± 4.80^Ab^	6.05	21.97	12.29 ± 3.94^Ba^	2.85	19.42	10.13 ± 3.71^Ba^

PAN, panavia F2; RXU, RelyX unicem; DUL, Duolink, SD = standard deviation. The values in each column and row with different superscript are significantly different at a 95% level of confidence. Differences within each row are shown in capital letters, and difference within each column are shown in small letters.

## Data Availability

The data are available upon request.

## References

[1] Wataha J. C. (2000). Biocompatibility of dental casting alloys: a review. *The Journal of Prosthetic Dentistry*.

[2] Luchetti C., Fratto G., Valeriani F. (2015). Cobalt-chromium alloys in dentistry: an evaluation of metal ion release. *The Journal of Prosthetic Dentistry*.

[3] Al Jabbari Y. S., Barmpagadaki X., Psarris I., Zinelis S. (2019). Microstructural, mechanical, ionic release and tarnish resistance characterization of porcelain fused to metal Co-Cr alloys manufactured via casting and three different CAD/CAM techniques. *Journal of Prosthodontic Research*.

[4] Li K. C., Prior D. J., Waddell J. N., Swain M. V. (2015). Comparison of the microstructure and phase stability of as-cast, CAD/CAM and powder metallurgy manufactured Co-Cr dental alloys. *Dental Materials*.

[5] Maroulakos G., Thompson G. A., Kontogiorgos E. D. (2019). Effect of cement type on the clinical performance and complications of zirconia and lithium disilicate tooth-supported crowns: a systematic review. Report of the committee on research in fixed prosthodontics of the American academy of fixed prosthodontics. *The Journal of Prosthetic Dentistry*.

[6] Edelhoff D., Özcan M. (2007). To what extent does the longevity of fixed dental prostheses depend on the function of the cement? working group 4 materials: cementation. *Clinical Oral Implants Research*.

[7] Pietursson B. E., Tan W. C., Tan K., Bragger U., Zwahein M., Lang N. P. (2008). A systematic review of the survival and complication rates of resin-bonded bridges after an observation period of at least 5 years. *Clinical Oral Implants Research*.

[8] Nima G., Ferreira P. V. C., Paula A. B. d., Consani S., Giannini M. (2017). Effect of metal primers on bond strength of a composite resin to nickel-chrome metal alloy. *Brazilian Dental Journal*.

[9] Yoshida K., Kamada K., Atsuta M. (1999). Adhesive primers for bonding cobalt-chromium alloy to resin. *Journal of Oral Rehabilitation*.

[10] Hill E. E. (2007). Dental cements for definitive luting: a review and practical clinical considerations. *Dental Clinics of North America*.

[11] Ferracane J. L., Stansbury J. W., Burke F. J. T. (2011). Self-adhesive resin cements—chemistry, properties and clinical considerations. *Journal of Oral Rehabilitation*.

[12] Weiser F., Behr M. (2015). Self-adhesive resin cements: a clinical review. *Journal of Prosthodontics*.

[13] Di Francescantonio M., de Oliveira M. T., Garcia R. N., Romanini J. C., da Silva N. R. F. A., Giannini M. (2010). Bond strength of resin cements to Co-Cr and Ni-Cr metal alloys using adhesive primers. *Journal of Prosthodontics*.

[14] Hattar S., Hatamleh M., Khraisat A., Al-Rabab’ah M. (2014). Shear bond strength of self-adhesive resin cements to base metal alloy. *The Journal of Prosthetic Dentistry*.

[15] Matsumura H., Yanagida H., Tanoue N., Atsuta M., Shimoe S. (2001). Shear bond strength of resin composite veneering material to gold alloy with varying metal surface preparations. *The Journal of Prosthetic Dentistry*.

[16] Choo S.-S., Huh Y.-H., Cho L.-R., Park C.-J. (2015). Effect of metal primers and tarnish treatment on bonding between dental alloys and veneer resin. *The Journal of Advanced Prosthodontics*.

[17] Abreu A., Loza M. A., Elias A., Mukhopadhyay S., Looney S., Rueggeberg F. A. (2009). Tensile bond strength of an adhesive resin cement to different alloys having various surface treatments. *The Journal of Prosthetic Dentistry*.

[18] Pinhero A., Silveira P. A. (2004). Effect of a metal primer on the bond strength of the resin—metal interface. *Journal of Applied Oral Science*.

[19] Shafiei F., Behroozibakhsh M, Abbasian A., Shahnavazi S. (2018). Bond strength of self-adhesive resin cement to base metal alloys having different surface treatments. *Dental Research Journal*.

[20] Dias de Souza G. M., Thompson V. P., Braga R. R. (2011). Effect of metal primers on microtensile bond strength between zirconia and resin cements. *The Journal of Prosthetic Dentistry*.

[21] Yun J.-Y., Ha S.-R., Lee J.-B., Kim S.-H. (2010). Effect of sandblasting and various metal primers on the shear bond strength of resin cement to Y-TZP ceramic. *Dental Materials*.

[22] Chen Y., Lu Z., Qian M. (2017). Chemical affinity of 10-methacryloyloxydecyl dihydrogen phosphate to dental zirconia: effects of molecular structure and solvents. *Dental Materials*.

[23] Yushida K. (2020). Effect of 10-metacryloyloxydecyl dihydrogen phosphate concentration in primers on bonding resin cements to zirconia. *Journal of Prosthodontics*.

[24] Yang B., Barloi A., Kern M. (2010). Influence of air-abrasion on zirconia ceramic bonding using an adhesive composite resin. *Dental Materials*.

[25] Abreu A., Loza M. A., Elias A., Mukhopadhyay S., Rueggeberg F. A. (2007). Effect of metal type and surface treatment on in vitro tensile strength of copings cemented to minimally retentive preparations. *The Journal of Prosthetic Dentistry*.

[26] Kern M., Thompson P. (1995). Durability of resin bonds to pure titanium. *Journal of Prosthodontics*.

[27] Padros R., Giner-Tarrida L., Herrero-Climent M., Punset M., Gil F. J. (2020). Corrosion resistance and ion release of dental prosthesis of CoCr obtained by CAD/CAM milling, casting, and laser sintering. *Metals*.

[28] Scherrer S. S., Cesar P. F., Swain M. V. (2010). Direct comparison of the bond strength results of the different test methods: a critical literature review. *Dental Materials*.

[29] Fonseca R. G., Martins S. B., de Oliveira Abi-Rached F., Dos Santos Cruz C. A. (2012). Effect of different airborne-particle abrasion/bonding agent combinations on the bond strength of a resin cement to a base metal alloy. *The Journal of Prosthetic Dentistry*.

